# Evaluation of Ganglion Cell–Inner Plexiform Layer Thickness in the Diagnosis of Preperimetric and Early Perimetric Glaucoma

**DOI:** 10.3390/jcm14197117

**Published:** 2025-10-09

**Authors:** Ilona Anita Kaczmarek, Marek Edmund Prost, Radosław Różycki

**Affiliations:** Department of Ophthalmology, Military Institute of Aviation Medicine, 54/56 Krasińskiego Street, 01-755 Warsaw, Poland

**Keywords:** preperimetric glaucoma, retinal ganglion cells, spectral-domain optical coherence tomography

## Abstract

**Background:** Optical coherence tomography (OCT) is the main diagnostic technology used to detect damage to the retinal ganglion cells (RGCs) in glaucoma. However, it remains unclear which OCT parameter demonstrates the best diagnostic performance for eyes with early, especially preperimetric glaucoma (PPG). We determined the diagnostic performance of ganglion cell–inner plexiform layer (GCIPL) parameters using spectral-domain OCT (SD-OCT) in primary open-angle preperimetric and early perimetric glaucoma and compared them with optic nerve head (ONH) and peripapillary retinal nerve fiber layer (pRNFL) parameters. **Methods:** We analyzed 101 eyes: 36 normal eyes, 33 with PPG, and 32 with early perimetric glaucoma. All patients underwent Topcon SD–OCT imaging using the Optic Disc and Macular Vertical protocols. The diagnostic abilities of the GCIPL, rim area, vertical cup-to-disc ratio (CDR), and pRNFL were assessed using the area under the receiver operating characteristic curve (AUC). **Results:** For PPG, the AUCs ranged from 0.60 to 0.63 (GCIPL), 0.82 to 0.86 (ONH), and 0.49 to 0.75 (pRNFL). For early perimetric glaucoma, the AUCs for GCIPL and pRNFL ranged from 0.81 to 0.88 and 0.57 to 0.91, respectively, whereas both ONH parameters demonstrated an AUC of 0.89. The GCIPL parameters were significantly lower than both ONH parameters in detecting preperimetric glaucoma (*p* < 0.05). For early perimetric glaucoma, comparisons between the AUCs of the best-performing mGCIPL parameters and those of the best-performing pRNFL and ONH parameters revealed no significant differences in their diagnostic abilities (*p* > 0.05). **Conclusions:** GCIPL parameters exhibited a diagnostic performance comparable to that of ONH and pRNFL parameters for early perimetric glaucoma. However, their ability to detect preperimetric glaucoma was significantly lower than the ONH parameters.

## 1. Introduction

Glaucoma is a progressive optic neuropathy and is the leading cause of irreversible blindness worldwide. This disease is characterized by pathological loss of retinal ganglion cells (RGCs), leading to the development of visual field (VF) defects [1]. The progressive degeneration of RGCs manifests as clinically detectable alterations in the optic nerve head (ONH) and thinning of the retinal nerve fiber layer (RNFL).

In the human retina, RGCs are distributed across the three inner retinal layers. The RNFL is formed by the axons of RGCs, the ganglion cell layer (GCL) contains the cell bodies of RGCs, and the dendrites of RGCs are located in the inner plexiform layer (IPL). Several histopathological studies have shed light on alterations in these inner retinal layers during the early stages of glaucoma [2,3,4,5]. Agostinone et al. reported that glaucoma-induced damage to RGCs results in changes in axons and somas, dendritic shrinkage, and synaptic loss, and that dendritic changes precede alterations in the other two cellular compartments [3]. Developing methods to quantify these early morphological changes in RGCs could facilitate the earlier detection of glaucoma.

Optical coherence tomography (OCT) is the primary diagnostic technology used to detect damage to the inner retinal layers, peripapillary retinal nerve fiber layer (pRNFL), and ONH in glaucoma. The 3D OCT-2000 FA (Topcon Corporation; Tokyo, Japan) is a spectral-domain optical coherence tomography (SD-OCT) device that includes an algorithm called Glaucoma Analysis-Macula, which measures and analyzes the thickness of the inner retinal layers of the macula. The algorithm separates the RNFL from the ganglion cell–inner plexiform layer (GCIPL) in the macular region. However, it cannot measure the thicknesses of the GCL and inner plexiform layer (IPL) separately. Thus, the algorithm enables the separate measurement of the mRNFL and GCIPL thicknesses, as well as the thickness of the ganglion cell complex (GCC), which comprises the mRNFL and GCIPL.

Recent studies have compared the diagnostic accuracies of GCIPL, pRNFL, and ONH in early glaucoma; however, their findings have been inconsistent [6,7,8,9,10,11]. Particularly, it remains unclear which OCT parameter demonstrates the best diagnostic performance for eyes with preperimetric glaucoma (PPG). Therefore, we determined the diagnostic ability of GCIPL parameters in eyes with early glaucoma and compared these parameters with ONH and peripapillary RNFL parameters.

## 2. Materials and Methods

We examined patients with primary open-angle glaucoma consecutively as they presented to the Department of Ophthalmology at the Military Institute of Aviation Medicine in Warsaw, Poland between October 2020 and August 2023, and recruited those who met the inclusion criteria. A normal control group was recruited from the healthy general population. Informed consent was obtained from all participants after explaining the nature of the procedures. This study was approved by the Hospital Ethics Committee and adhered to the tenets of the Declaration of Helsinki.

A total of 101 eyes of 101 Caucasian subjects were included in the study: 33 eyes with PPG, 32 eyes with early perimetric glaucoma (EPG), and 36 normal control eyes. When both eyes were eligible, one eye was randomly selected from each participant.

All participants underwent a comprehensive ophthalmic examination, including a review of medical history, measurement of refraction, best-corrected visual acuity (BCVA), and intraocular pressure (IOP) using Goldmann applanation tonometry, slit-lamp biomicroscopy with dilated fundus examination using a 78 D lens, ultrasound pachymetry, axial length measurement using an optical biometer Zeiss IOL Master 500 (Carl Zeiss Meditec; Jena, Germany), visual field testing, and SD–OCT imaging with the 3D OCT-2000 FA (Topcon Corporation; Tokyo, Japan). All tests were conducted over 2 weeks.

VF testing was conducted for each eye using standard automated perimetry with a Humphrey Field Analyzer 3 (HFA) and Swedish Interactive Threshold Algorithm Standard (SITA-Standard) 30-2 test (Carl Zeiss Meditec; Jena, Germany) [12]. For inclusion in the study, all eyes required high-quality standard automated perimetry results. A VF test was considered reliable if fixation losses and false-negative error rates were less than 20%, and false-positive response rates were less than 15% [12,13]. All participants underwent more than one HFA. Only the results of the second reliable HFA test were used in the analysis to minimize the learning effect.

An HFA result was defined as normal when the mean deviation (MD) and pattern standard deviation (PSD) were within the 95% confidence limits of the normative reference and the glaucoma hemifield test result was within normal limits [12].

Glaucomatous VF defects were defined as a cluster of three or more non-edge points with a probability of less than 5%, including at least one point with a probability of less than 1% in the pattern deviation probability plot or a glaucoma hemifield test result outside the normal limits. Any of the aforementioned criteria, if repeatable, were considered sufficient evidence of a glaucomatous VF defect [12].

To be included in the study, all subjects were required to meet the following criteria: age ≥ 18 years, BCVA of at least 6/12 on the Snellen chart, refractive error within ±5 diopters (D) sphere and ±3 D cylinder, and the presence of an open anterior chamber angle on gonioscopy.

Exclusion criteria included a history of intraocular surgery other than uncomplicated cataract surgery, presence of secondary glaucoma, coexisting retinal (particularly macular) or neurological diseases other than glaucoma that could confound the results, unreliable Humphrey VF test results, or unacceptable OCT image quality (TopQ Image Quality Index <30). Patients with malignancies, those receiving corticosteroids or cytostatics, and pregnant women were excluded.

Participants’ eyes were categorized into three groups: normal, PPG, and EPG. The eligibility criteria for each group are outlined below:

The inclusion criteria for the control group (36 eyes) were as follows: healthy subjects with no first-degree family history of glaucoma; no previous intraocular surgery; IOP < 21 mmHg and no history of elevated IOP; normal VF test results; and non-glaucomatous ONH appearance on slit-lamp biomicroscopy, defined as an intact neuroretinal rim without peripapillary hemorrhage and no peripapillary RNFL defects on red-free slit-lamp biomicroscopy.

Eyes with PPG (33 eyes) were defined as those with normal VF and characteristic glaucomatous ONH changes, such as focal or diffuse neuroretinal rim thinning, localized notching, or RNFL defects. Only patients with unilateral PPG were included in the study. Unilateral PPG was defined as the presence of the aforementioned PPG criteria in one eye, and perimetric glaucoma in the other.

Eyes with EPG (32 eyes) were defined as those exhibiting characteristic glaucomatous ONH changes (focal or diffuse neuroretinal rim thinning, localized notching, or RNFL defects) and early perimetric glaucomatous VF damage, based on the Hodapp–Parrish–Anderson classification (mean deviation ≥ −6 dB). Only patients taking anti-glaucoma medications (eye drops) were included in the PPG and EPG groups. Therefore, IOP was not used as an inclusion criterion in the glaucoma group.

SD-OCT imaging was performed using a 3D OCT-2000 FA Plus system (software version 8.39; Topcon Corporation). The 3D Macula Vertical protocol was used to assess the GCIPL parameters, whereas the 3D disc protocol was used to assess the ONH and pRNFL parameters. OCT was performed on each qualifying eye after pupil dilation with 1% tropicamide. Only high-quality scans with a TopQ Image Quality index of at least 30 and without RNFL discontinuity, segmentation error, or blinking artifacts were included in the analysis.

The 3D Macula Vertical protocol was designed to measure mRNFL, GCIPL, and GCC thickness using a 7 × 7 mm scanning area centered on the fovea with a scan density of 512 × 128 (128 horizontal scan lines comprising 512 A-scans each). In this study, the average total, superior, and inferior GCIPL thicknesses were analyzed.

The ONH parameters generated by the 3D Disc 512 × 128 protocol (6 × 6 mm scanning area) were evaluated in this study, including the optic disc area, rim area, and vertical cup-to-disc ratio (CDR). The RNFL parameters calculated using the same protocol included the average total RNFL thickness and quadrant specific RNFL thickness (superior, inferior, nasal, and temporal).

Statistical analyses were performed using Statistica version 13.3 (StatSoft Inc., Tulsa, OK, USA) and MedCalc version 20.015 (MedCalc Software Ltd., Ostend, Belgium). The Shapiro–Wilk test was used to assess the normality of the data distribution. Descriptive statistics included mean and standard deviation (SD) for normally distributed variables and median, first quartile, and third quartile values for non-normally distributed variables. Differences among the normal control, preperimetric glaucoma, and early glaucoma groups were evaluated using Fisher’s exact test, the Kruskal–Wallis test, or one-way analysis of variance (ANOVA) followed by Tukey’s multiple comparison test.

The area under the receiver operating characteristic curve (AUC) was used to evaluate the ability of each SD–OCT software-derived parameter to discriminate glaucomatous eyes from control eyes. The receiver operating characteristic (ROC) curve illustrates the relationship between sensitivity and 1-specificity for a given diagnostic test. Sensitivity at a fixed specificity of 95% was calculated for each parameter. Confidence intervals (CIs) for AUCs were obtained using the bootstrap resampling method (*n* = 1000 resamples). The Z-test was used to compare AUCs for statistical significance [14,15]. Statistical significance was set at *p* < 0.05.

## 3. Results

Of the 146 patients initially enrolled, 34 were excluded due to unreliable VF results, and 11 patients (seven with glaucoma and four controls) were excluded due to poor-quality OCT scans (TopQ Image Quality Index < 30). Ultimately, 101 eyes (65 glaucoma patients and 36 controls) were included in the analysis.

The demographic and clinical characteristics of each group are summarized in Table 1. No significant differences were observed between the groups in terms of sex, age, axial length, spherical equivalent, or IOP. As expected, statistically significant differences were observed in MD and PSD between the groups.

Table 2 presents the mean values of the diagnostic parameters in normal controls and eyes with preperimetric and early perimetric glaucoma. Notably, no significant differences were noted in the disc area among the three groups. However, the rim area and vertical CDR differed significantly between the control and glaucoma groups. The early perimetric glaucoma group exhibited significantly thinner pRNFL and GCIPL than the normal controls, except for temporal pRNFL thickness. Patients with preperimetric glaucoma showed significantly thinner pRNFL in the superior and inferior quadrants than normal controls. None of the GCIPL parameters differed significantly between the control and preperimetric glaucoma groups (*p* > 0.05).

The diagnostic ability of each parameter, as assessed by the AUC, is presented in Table 3. The best-performing parameters for distinguishing normal controls from eyes with preperimetric glaucoma were the vertical CDR (AUC = 0.86) and rim area (AUC = 0.82). When distinguishing preperimetric glaucoma, GCIPL parameters yielded significantly lower AUCs compared with the rim area and vertical CDR (*p* < 0.05).

The AUCs of macular GCIPL (mGCIPL) parameters for detecting early perimetric glaucoma ranged from 0.81 to 0.88. Comparisons between the AUCs of the best-performing mGCIPL parameters and those of the best-performing pRNFL and ONH parameters revealed no significant differences in their diagnostic abilities (AUC of mGCIPL inferior (0.88) vs. AUC of pRNFL superior (0.91), *p* = 0.49; AUC of mGCIPL inferior (0.88) vs. AUC of Rim Area (0.89), *p* = 0.89; AUC of mGCIPL inferior (0.88) vs. AUC of Vertical CDR (0.89), *p* = 0.80).

Additionally, no significant differences were observed between the AUCs of pRNFL superior (0.91) and Rim Area (0.89) in early perimetric glaucoma (*p* = 0.73). However, it should be emphasized that the Rim Area parameter demonstrated the highest sensitivity at 95% specificity (81.3%) and therefore may be more suitable for diagnosing early perimetric glaucoma.

Notably, no single GCIPL parameter demonstrated a significantly higher AUC than the others for diagnosing pre-perimetric or early perimetric glaucoma (*p* > 0.05 for all GCIPL parameter comparisons).

## 4. Discussion

Despite numerous studies evaluating the role of macular GCIPL thickness in glaucoma detection [16], relatively few have compared the diagnostic ability of mGCIPL and ONH parameters, specifically in early and particularly in preperimetric glaucoma [6,7,10,11]. The results of some of these studies differ from one another [6,10]; therefore, the utility of mGCIPL thickness in diagnosing preperimetric glaucoma and its comparison with ONH parameters remain to be fully elucidated. The present study evaluated the ability of GCIPL parameters to distinguish healthy eyes from those with preperimetric or early perimetric glaucoma and compared the diagnostic performance of GCIPL parameters with that of ONH and pRNFL thickness using the Topcon 3D OCT-2000 FA system.

In all previously published studies, preperimetric glaucoma was defined by a normal VF, the presence of RNFL defects, and glaucomatous optic disc changes observed on fundus examination. However, the specific criteria for defining glaucomatous optic disc damage in preperimetric glaucoma vary across studies [6,17]. Begum et al. defined the optic disc as glaucomatous based on the presence of focal or diffuse neuroretinal rim thinning, localized notching, or RNFL defects [6]. Similarly, Edlinger et al. included advanced optic disc excavation and increased cup-to-disc ratio as indicators of glaucomatous damage [17]. Furthermore, Deshpande et al. included an IOP greater than 21 mmHg as a criterion for diagnosing preperimetric glaucoma [11].

In the present study, we defined PPG using criteria similar to those described by Begum et al. [6]. However, we included only subjects with unilateral PPG in the PPG group. Unilateral PPG was defined as the presence of PPG in one eye and perimetric glaucoma in the other eye. This restrictive diagnostic criterion was used to reduce the likelihood of false positive results [18].

Our findings demonstrated that the diagnostic ability of GCIPL parameters in preperimetric glaucoma was significantly lower than that of ONH parameters, based on comparisons of the AUC. Similar results have been reported by Begum et al. [6]. One possible explanation for this observation is the distribution of different types of RGCs within the retina. Previous studies have indicated that parasol RGCs—also known as “M” cells, which comprise the magnocellular visual pathway—are particularly susceptible to elevated IOP and are the first to be damaged in the early stages of glaucoma [19,20]. These large “M” cells are predominantly located in the peripheral retina and are virtually absent in the macular region [21,22]. Because GCIPL parameters are measured within the macular region using OCT, early damage to peripherally located parasol RGCs may not be detected using this algorithm. Therefore, in preperimetric glaucoma, GCIPL values may remain within normal limits. In contrast, the rim area, a parameter derived from ONH analysis, reflects the integrity of the axons of all RGCs in the retina. Therefore, the detection rate for ganglion cell damage in preperimetric glaucoma is expected to be higher with ONH parameters, such as rim area, than with GCIPL parameters.

In contrast to our findings, Kim et al. observed that the diagnostic performance of GCIPL parameters was comparable to that of ONH parameters in preperimetric glaucoma [7]. Their results differed from ours, most likely because of differences in the definition of preperimetric glaucoma. Kim et al. included eyes with PPG that demonstrated documented evidence of progression (e.g., narrowing of the neuroretinal rim) assessed at least 6 months before enrollment [7]. In our study, progressive glaucomatous optic disc changes were not required as an inclusion criterion. Consequently, Kim et al. might have included patients with more advanced glaucomatous changes than those included in our study. Moreover, optic nerve head parameters may vary according to race and ethnicity [23]. Kim et al. enrolled only Korean participants, whereas our study included only Caucasian subjects [7].

Among the pRNFL parameters, we found that average, inferior, and superior RNFL thicknesses yielded higher AUCs for diagnosing preperimetric glaucoma than nasal or temporal RNFL thicknesses. These results are consistent with those of previous studies [10,24]. Although the best-performing GCIPL parameter had a lower AUC than the best-performing RNFL parameter, the difference was not statistically significant (AUC for the inferior GCIPL vs. the superior pRNFL, *p* = 0.08). Table 4 presents a comparison of the AUC and sensitivity at 95% specificity of OCT parameters for diagnosing preperimetric glaucoma as reported in other studies.

Early perimetric glaucoma is diagnosed based on the presence of characteristic glaucomatous structural changes in the optic disc and pRNFL defects accompanied by early glaucomatous VF loss [25]. In our study, the MD in the early perimetric glaucoma group was −3.10 dB (interquartile range: −4.39, −1.37). We found that the diagnostic ability of the GCIPL parameters in early perimetric glaucoma was high and comparable to that of the ONH and pRNFL parameters, based on comparisons of their best-performing AUCs. These findings are consistent with those of previous studies [8,24]. Table 5 provides a comparison of the AUC, sensitivity, and specificity of OCT parameters for the diagnosis of early perimetric glaucoma reported in previous studies.

The present study had several limitations. The PPG group was defined as the presence of PPG in one eye (included in the analysis) and perimetric glaucoma in the other. This approach was used to minimize the inclusion of false-positive cases, that is, eyes with normal VF that did not truly have glaucomatous damage. However, we may have excluded patients with bilateral preperimetric glaucoma by applying these stringent diagnostic criteria.

Additionally, we did not evaluate parameters that reflect the volumetric loss of macular retinal ganglion cells, specifically the global loss volume (GLV) and focal loss volume (FLV). The OCT system used in this study (3D OCT-2000 FA; Topcon Corporation) did not include the GLV or FLV in retinal ganglion cell assessment. Arintawati et al. reported that in early glaucoma with mild VF loss, GLV exhibited superior diagnostic capability compared with RNFL parameters [26]. Another limitation was the relatively small sample size. Larger population-based studies are required to validate and generalize these findings.

## 5. Conclusions

The diagnostic ability of GCIPL parameters obtained using the 3D OCT-2000 system was similar to ONH and pRNFL indices in established early perimetric glaucoma. However, no consensus exists on the most effective OCT parameters for diagnosing preperimetric glaucoma. Our findings indicate that GCIPL parameters have a significantly lower diagnostic ability than ONH parameters for preperimetric glaucoma. Further clinical research is warranted to elucidate the role of GCIPL measurements in the detection of early glaucomatous damage.

## Figures and Tables

**Table 1:** Demographic and clinical characteristics of study participants.

Parameters	Normal	Preperimetric Glaucoma	Early Perimetric Glaucoma	*p*1	*p*2	*p*3
No. participants	36	33	32			
Sex (male/female)	9/27	11/22	10/22	0.31	0.38	0.53
Age (years)	63.56 ± 7.68	65.82 ± 8.23	66.94 ± 8.79	0.51	0.23	0.85
Axial length (mm)	23.12 ± 0.82	23.16 ± 0.85	23.02 ± 1.00	0.98	0.91	0.81
SE (D)	−0.05 ± 1.25	0.14 ± 1.72	−0.16 ± 1.89	0.88	0.96	0.74
IOP (mmHg)	16.08 ± 2.91	15.58 ± 3.21	14.91 ± 2.97	0.78	0.27	0.65
CCT (μm)	546.69 ± 26.41	526.94 ± 37.75	523.38 ± 31.41	0.04	0.01	0.90
MD (dB)	0.15 (−0.62, 0.67)	−0.42 (−1.06, 0.69)	−3.10 (−4.39, −1.37)	0.87	<0.001	<0.001
PSD (dB)	1.68 (1.51, 1.92)	1.77 (1.51, 1.90)	3.74 (2.76, 5.28)	1	<0.001	<0.001

Data are presented as mean ± SD or median (first quartile, third quartile). *p*1: Control vs. preperimetric glaucoma, *p*2: control vs. early perimetric glaucoma, *p*3: preperimetric vs. early perimetric glaucoma. Statistical tests: Fisher’s exact test for sex distribution; one-way analysis of variance (ANOVA) or Kruskal–Wallis test for continuous variables. SE, spherical equivalent; IOP, intraocular pressure; CCT, central corneal thickness; MD, mean deviation; PSD, pattern standard deviation.

**Table 2:** Distribution of diagnostic parameters among study groups.

Parameters	Normal	Preperimetric Glaucoma	Early Perimetric Glaucoma	*p*1	*p*2	*p*3
ONH						
Disc area (mm^2^)	2.12 ± 0.30	2.23 ± 0.32	2.22 ± 0.50	0.46	0.50	0.99
Rim area (mm^2^)	1.50 ± 0.26	1.10 ± 0.33	0.92 ± 0.40	<0.01	<0.01	0.08
Vertical CDR	0.51 ± 0.15	0.70 ± 0.11	0.76 ± 0.17	<0.01	<0.01	0.17
pRNFL thickness (μm)						
Average	96.28 ± 7.48	90.52 ± 10.59	79.28 ± 11.91	0.06	<0.01	<0.01
Superior	115.31 ± 11.50	103.18 ± 13.56	87.44 ± 17.24	<0.01	<0.01	<0.01
Inferior	119.53 ± 11.29	107.91 ± 14.41	94.50 ± 18.86	<0.01	<0.01	0.04
Nasal	81.47 ± 13.94	79.58 ± 19.09	70.97 ± 14.44	0.97	0.02	0.23
Temporal	68.64 ± 8.91	71.45 ± 14.98	64.00 ± 13.32	0.63	0.80	0.53
mGCIPL (μm)						
Average	67.22 ± 4.56	64.91 ± 6.09	59.09 ± 6.03	0.22	<0.01	<0.01
Superior	67.08 ± 4.71	65.52 ± 6.36	59.44 ± 6.80	0.54	<0.01	<0.01
Inferior	67.58 ± 5.23	64.45 ± 6.62	58.66 ± 6.36	0.10	<0.01	<0.01

Data are expressed as mean ± SD. Statistical analysis: ANOVA. *p*1 = Control vs. preperimetric glaucoma, *p*2 = control vs. early perimetric glaucoma, *p*3 = preperimetric vs. early perimetric glaucoma. ONH, optic nerve head; CDR, cup-to-disc ratio; pRNFL, peripapillary retinal nerve fiber layer; mGCIPL, macular ganglion cell–inner plexiform layer.

**Table 3:** AUC and sensitivity at 95% specificity of SD–OCT parameters for diagnosing preperimetric and early perimetric glaucoma.

	Preperimetric Glaucoma			Early Perimetric Glaucoma		
Parameters	AUC (95% CI)	Sensitivity at 95% Specificity (%)	*p*	AUC (95% CI)	Sensitivity at 95% Specificity (%)	*p*
ONH						
Rim area	0.82 (0.72–0.92)	45.5	<0.05	0.89 (0.79–0.98)	81.3	<0.05
Vertical CDR	0.86 (0.77–0.95)	42.4	<0.05	0.89 (0.80–0.98)	71.9	<0.05
pRNFL thickness						
Average	0.68 (0.55–0.81)	26.7	<0.05	0.88 (0.80–0.96)	59.4	<0.05
Superior	0.75 (0.64–0.86)	30.3	<0.05	0.91 (0.84–0.96)	68.8	<0.05
Inferior	0.73 (0.62–0.85)	27.3	<0.05	0.86 (0.77–0.95)	53.1	<0.05
Nasal	0.56 (0.42–0.70)	12.1	0.38	0.70 (0.58–0.83)	18.8	<0.05
Temporal	0.49 (0.35–0.63)	6.1	0.84	0.57 (0.43–0.72)	25.0	0.32
mGCIPL						
Average	0.63 (0.50–0.76)	24.2	0.06	0.86 (0.77–0.95)	49.4	<0.05
Superior	0.60 (0.46–0.73)	21.2	0.17	0.81 (0.71–0.92)	49.1	<0.05
Inferior	0.63 (0.50–0.77)	24.2	0.05	0.88 (0.79–0.96)	49.6	<0.05

AUC, area under the receiver operating characteristic curve; CI, confidence interval; ONH, optic nerve head; CDR, cup-to-disc ratio; pRNFL, peripapillary retinal nerve fiber layer; mGCIPL, macular ganglion cell–inner plexiform layer.

**Table 4:** Comparison of the AUC and sensitivity at 95% specificity of OCT parameters in studies on the diagnosis of preperimetric glaucoma.

Study	Parameters	AUC (95% CI)	Sensitivity at 95% Specificity (%)	*p*
Begum, V.U. et al. [6]	ONH			
	Rim area	0.85 (0.73–0.93)	48	ND
	Vertical CDR	0.92 (0.82–0.99)	76	ND
	pRNFL thickness			
	Average	0.79 (0.64–0.88)	24	ND
	Superior	0.79 (0.64–0.91)	10	ND
	Inferior	0.80 (0.64–0.90)	38	ND
	Nasal	0.58 (0.40–0.74)	14	ND
	Temporal	0.50 (0.35–0.62)	5	ND
	mGCIPL			
	Average	0.59 (0.43–0.74)	5	ND
	Superior	0.63 (0.44–0.75)	5	ND
	Inferior	0.58 (0.40–0.73)	0	ND
Tai, T.Y. et al. [10]	ONH			
	Rim area	0.801	38.5	ND
	Vertical CDR	0.772	41.0	ND
	pRNFL thickness			
	Average	0.819	38.5	ND
	Superior	0.783	53.8	ND
	Inferior	0.821	35.9	ND
	Nasal	0.606	10.3	ND
	Temporal	0.643	17.9	ND
	mGCIPL			
	Average	0.745	15.4	ND
	Superior	0.699	15.4	ND
	Inferior	0.751	25.6	ND
Current study	ONH			
	Rim area	0.82 (0.72–0.92)	45.5	<0.05
	Vertical CDR	0.86 (0.77–0.95)	42.4	<0.05
	pRNFL thickness			
	Average	0.68 (0.55–0.81)	26.7	<0.05
	Superior	0.75 (0.64–0.86)	30.3	<0.05
	Inferior	0.73 (0.62–0.85)	27.3	<0.05
	Nasal	0.56 (0.42–0.70)	12.1	0.38
	Temporal	0.49 (0.35–0.63)	6.1	0.84
	mGCIPL			
	Average	0.63 (0.50–0.76)	24.2	0.06
	Superior	0.60 (0.46–0.73)	21.2	0.17
	Inferior	0.63 (0.50–0.77)	24.2	0.05

AUC, area under the receiver operating characteristic curve; CI, confidence interval; ONH, optic nerve head; CDR, cup-to-disc ratio; pRNFL, peripapillary retinal nerve fiber layer; mGCIPL, macular ganglion cell-inner plexiform layer; ND, no data.

**Table 5:** Comparison of the AUC, sensitivity, and specificity of OCT parameters in studies on the diagnosis of early perimetric glaucoma.

Study	Parameters	AUC (95% CI)	Sensitivity (%)	Specificity (%)	*p*
Mwanza, J.C. et al. [8]	ONH				
	Rim area	0.91	67.2	95.9	ND
	Vertical CDR	0.962	87.9	90.9	ND
	pRNFL thickness				
	Average	0.936	81	92.9	ND
	Superior	0.933	79.3	81.8	ND
	Inferior	0.939	93.1	98.9	ND
	Nasal	ND	ND	ND	ND
	Temporal	ND	ND	ND	ND
	mGCIPL				
	Average	0.935	87.9	86.8	ND
	Superior	0.875	72.4	87.9	ND
	Inferior	0.918	75.9	91.9	ND
Jeoung, J.W. et al. [9]	ONH				
	Rim area	0.855	61.0	86.6	ND
	Vertical CDR	0.721	57.9	84.9	ND
	pRNFL thickness				
	Average	0.897	50.0	96.6	ND
	Superior	0.815	43.3	97.5	ND
	Inferior	0.890	61.6	94.6	ND
	Nasal	0.664	12.8	99.2	ND
	Temporal	0.673	18.3	99.2	ND
	mGCIPL				
	Average	0.817	50.6	50.6	ND
	Superior	0.714	37.8	92.4	ND
	Inferior	0.817	57.9	57.9	ND
Current study	ONH				
	Rim area	0.89 (0.79-0.98)	81.3	95	<0.05
	Vertical CDR	0.89 (0.80-0.98)	71.9	95	<0.05
	pRNFL thickness				
	Average	0.88 (0.80–0.96)	59.4	95	<0.05
	Superior	0.91 (0.84–0.96)	68.8	95	<0.05
	Inferior	0.86 (0.77–0.95)	53.1	95	<0.05
	Nasal	0.70 (0.58–0.83)	18.8	95	<0.05
	Temporal	0.57 (0.43–0.72)	25.0	95	0.32
	mGCIPL				
	Average	0.86 (0.77–0.95)	49.4	95	<0.05
	Superior	0.81 (0.71–0.92)	49.1	95	<0.05
	Inferior	0.88 (0.79–0.96)	49.6	95	<0.05

AUC, area under the receiver operating characteristic curve; CI, confidence interval; CDR, cup-to-disc ratio; ONH, optic nerve head; pRNFL, peripapillary retinal nerve fiber layer; mGCIPL, macular ganglion cell-inner plexiform layer; ND, no data.

## Data Availability

The original data presented in this study are included in the article. Further inquiries can be directed to the corresponding author.

## Abbreviations

The following abbreviations are used in this manuscript:

GCIPL	Ganglion cell–inner plexiform layer
SD–OCT	Spectral-domain optical coherence tomography
ONH	Optic nerve head
AUC	Area under the receiver operating characteristic curve
ROC	Receiver operating characteristic curve
ANOVA	Analysis of variance
pRNFL	Peripapillary retinal nerve fiber layer
VF	Visual field
GCL	Ganglion cell layer
RGC	Retinal ganglion cells
IPL	Inner plexiform layer
GCC	Ganglion cell complex
IOP	Intraocular pressure
BCVA	Best-corrected visual acuity
EPG	Early perimetric glaucoma
PPG	Preperimetric glaucoma
HFA	Humphrey Field Analyzer 3
PSD	Pattern standard deviation
MD	Mean deviation
CDR	Cup-to-disc ratio
SD	Standard deviation
CI	Confidence interval
